# Surface Photochemistry: 3,3′-Dialkylthia and Selenocarbocyanine Dyes Adsorbed onto Microcrystalline Cellulose

**DOI:** 10.3390/ijms13010596

**Published:** 2012-01-09

**Authors:** Luís F. Vieira Ferreira, Diana P. Ferreira, Paulo Duarte, A. S. Oliveira, E. Torres, I. Ferreira Machado, P. Almeida, Lucinda V. Reis, Paulo F. Santos

**Affiliations:** 1 Molecular Physical Chemistry Center, and IN-Institute of Nanoscience and Nanotechnology, Instituto Superior Técnico, Technical University of Lisbon, Av. Rovisco Pais, 1049-001 Lisboa, Portugal; E-Mails: diana.ferreira@ist.utl.pt (D.P.F.); paulo.duarte@ist.utl.pt (P.D.); asoliveira@ist.utl.pt (A.S.O.); erica.torres@ist.utl.pt (E.T.); ilferreiramachado@ist.utl.pt (I.F.M.); 2 School of Technology and Business, Polytechnic Institute of Portalegre, Lugar da Abadessa, Apt 148, 7301-901 Portalegre, Portugal; 3 CICS - Health Sciences Research Center, Beira Interior University, Av. Infante D. Henrique, 6200-506 Covilhã, Portugal; E-Mail: paulo.almeida@ubi.pt; 4 Department of Chemistry and Chemistry Center, Trás-os-Montes e Alto Douro University, Apartado 1013, 5001-801 Vila Real, Portugal; E-Mails: lucinda.reis@utad.pt (L.V.R.); psantos@utad.pt (P.F.S.)

**Keywords:** surfaces and nanocavities, thia and selenocarbocyanine dyes, lifetime distribution analysis, absolute quantum yields of fluorescence emission, singlet oxygen formation quantum yield

## Abstract

In this work, thia and selenocarbocyanines with *n*-alkyl chains of different length, namely with methyl, ethyl, propyl, hexyl and decyl substituents, were studied in homogeneous and heterogeneous media for comparison purposes. For both carbocyanine dyes adsorbed onto microcrystalline cellulose, a remarkable increase in the fluorescence quantum yields and lifetimes were detected, when compared with solution. Contrary to the solution behaviour, where the increase in the *n*-alkyl chains length increases to a certain extent the fluorescence emission Φ_F_ and τ_F_, on powdered solid samples a decrease of Φ_F_ and τ_F_ was observed. The use of an integrating sphere enabled us to obtain absolute Φ_F_’s for all the powdered samples. The main difference for liquid homogeneous samples is that the increase of the alkyl chain strongly decreases the Φ_F_ values, both for thiacarbocyanines and selenocarbocyanines. A lifetime distribution analysis for the fluorescence of these dyes adsorbed onto microcrystalline cellulose, evidenced location on the ordered and crystalline part of the substrate, as well as on the more disordered region where the lifetime is smaller. The increase of the *n*-alkyl chains length decreases the photoisomer emission for the dyes adsorbed onto microcrystalline cellulose, as detected for high fluences of the laser excitation, for most samples.

## 1. Introduction

Carbocyanine dyes are one of the most well studied families of dyes due to their use as sensitizers in photodynamic therapy, as laser dyes and optical data storage materials, in light harvesting systems, among many other uses [1–3]. These applications justify the enormous interest in the study of their photophysical and photochemical properties [4–9].

In solution and without bulky substituents in the polymethine chain, carbocyanine dyes adopt an all-*trans* configuration (N ground state configuration) [3,9]. From the first excited single state ^1^N the dye may isomerise to a mono-*cis* configuration, the P ground state photoisomer. This P photoisomer is emissive, and its fluorescence is red shifted in relation to the ^1^N emission. Aramendía *et al*. [3] present a clear scheme that takes all these photophysical processes into account, and also considers a twisted excited state intermediate, which decays to the N or P ground state isomers.

Microcrystalline cellulose is a purified form of cellulose, obtained from the natural polymer after a severe acid hydrolysis treatment in which the amorphous regions are preferentially attacked and transformed into a crystalline residue [10]. The final polymer has a high degree of crystallinity, the hydroxyl groups of one chain forming hydrogen bonds with the neighbouring chains, thus ensuring the rigidity of the matrix. By the use of ethanol, cellulose undergoes considerable swelling which results from the formation of new hydrogen bonds between the solvent molecules and the hydroxyl groups of cellulose. In this way, dye molecules can penetrate into sub microscopic pores and after solvent removal, ethanol cellulose hydrogen bonds are replaced with cellulose-cellulose and cellulose-dye-cellulose hydrogen bonds, the dye molecules becoming rigidly trapped between cellulose chains [11–15].

We have shown that many dye molecules such as rhodamines or oxazines and several ketones [11,12] are able to penetrate into the polymer matrix resulting in a decrease in the non-radiative pathways for deactivation of these dyes which can be used as entrapped excited probes.

In previous papers [5,6] we studied the emission of carbocyanine, thiacarbocyanine and oxocarbocyanine dyes, either in solution or when adsorbed onto microcrystalline cellulose. The observed emissions were dependent on the loadings of the adsorbed dye, on the fluences of the laser used to create the excited state populations, on the type of aggregation exhibited by the dye and also on the amount of water adsorbed on the cellulose surface, *i.e.*, the sample’s humidity degree.

**Figure 1:**
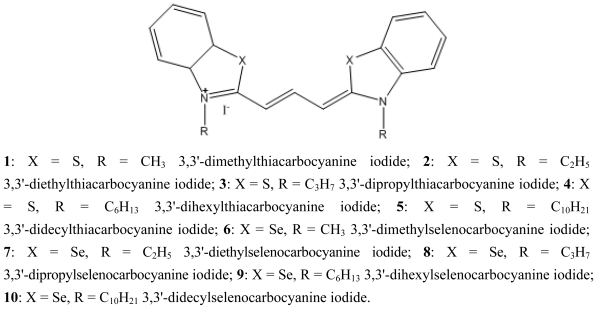
Structures of 3,3′-dialkylthia and selenocarbocyanine dyes.

The effect of the length of the alkyl chain on the photophysics of thiacarbocyanines adsorbed onto microcrystalline cellulose suggested the decrease of fluorescence quantum yield for longer substituents [15], contrary to the solution behaviour [4]. In this work we present a study of thia and selenocarbocyanines with *n*-alkyl chains of different length, namely with methyl, ethyl, propyl, hexyl and decyl substituents (please see Figure 1), both for homogeneous and heterogeneous media.

Both low and high fluences of the excitation laser were taken into account to study the behaviour of these dyes in solution and adsorbed onto powdered solid samples.

## 2. Results and Discussion

### 2.1. Ground State Diffuse Reflectance and Infrared (FTIR) Absorption Spectra

**Figure 2:**
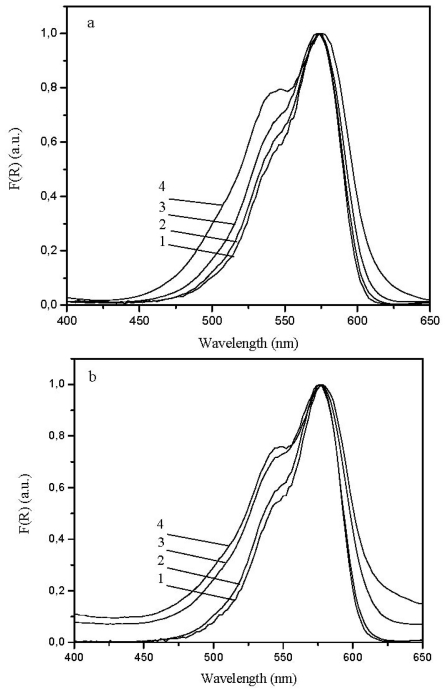
(**a**) Remission function values for 3,3′-diethylselenocarbocyanine iodide adsorbed onto microcrystalline cellulose for (1) 5 × 10^−8^, (2) 2 × 10^−7^, (3) 1 × 10^−6^ and (4) 5 × 10^−6^ mol of dye per gram of substrate. All data are normalized to the maximum value for the remission function; (**b**) Remission function values for 3,3′-diehexylselenocarbocyanine iodide adsorbed onto microcrystalline cellulose for (1) 5 × 10^−8^, (2) 2 × 10^−7^, (3) 1 × 10^−6^ and (4) 5 × 10^−6^ mol of dye per gram of substrate. All data are normalized to the maximum value for the remission function.

Figure 2a plots F(*R*)dye, the Kubelka Munk remission function *vs*. wavelength for 3,3′-diethylselenocarbocyanine adsorbed onto microcrystalline cellulose (ethanol was used for sample preparation, solvent which efficiently swells cellulose), at various loadings of the dye, normalised at the maximum absorption. At low loadings the absorption curve peaks at 574 nm, with a shoulder at about 542 nm and resemble the ones obtained in ethanolic solution (concentration ~1 × 10^−6^ M), apart from a *ca.* 4 nm shift in the solid powdered sample, as previously observed for 2,2′-diethylcarbocyanine [5]. As the loading of the dye increases, the shoulder at ~540 nm increases, indicating the formation of H aggregates. This behaviour is similar to the one observed for several carbocyanine dyes [5], thiacarbocyanine dyes [15] and oxocarbocyanine dyes [6]. Figure 2b presents very similar data for 3,3′-dihexylselenocarbocyanine adsorbed onto microcrystalline cellulose, where a H aggregate formation was also detected. All other dyes under study, *i.e.*, with methyl, propyl and decyl substituents provided similar behaviour and only sandwich type aggregates were formed in air equilibrated samples. These air equilibrated samples contain a certain degree of moisture (the laboratory usually presents about 60% degree of humidity), and cellulose adsorbs some water due to hydrogen bond formation with the numerous hydroxyl groups that exist in this natural polymer. As reported before [10], in well dried samples, the degree of dye aggregation decreases.

For thiacarbocyanines with methyl, ethyl, propyl, hexyl and decyl substituents, all dyes adsorbed onto microcrystalline cellulose provided very similar data (not presented in this paper) as the ones presented in Figure 2a,b.

The main conclusion from all these ground state absorption data is that the increase in the alkyl length of the 3,3′-substituents does not affect aggregation substantially, in air equilibrated samples in the range of concentrations under study.

### 2.2. Laser Induced Fluorescence of Air Equilibrated Samples

**Figure 3:**
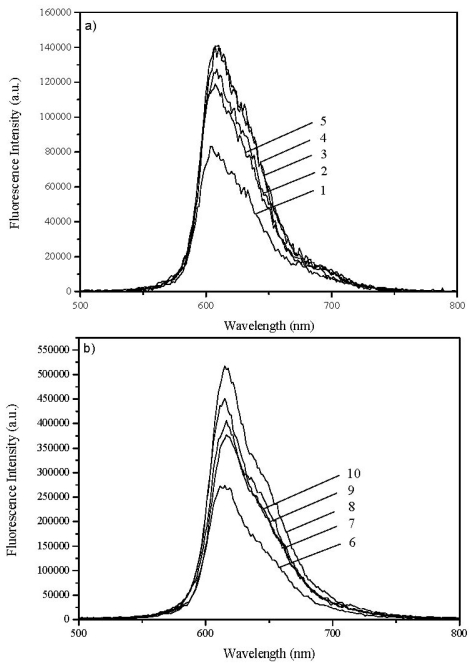
Fluorescence emission spectra of dyes: (**a**) 1, 2, 3, 4, 5 and (**b**) 6, 7, 8, 9, 10 in chloroform. The concentration was 1 × 10^−6^ M and the excitation wavelength used was 337 nm in all cases.

Figure 3 shows the laser induced fluorescence of 3,3′-dialkylthiacarbocyanine and 3,3′-dialkylselenocarbocyanine in chloroform for the alkyl substituents of the dye used in this work. The concentration of the dye is constant and is 1 × 10^−6^ M in all cases. One can observe small variations in Φ_F_, depending on the alkyl chain length of the dye under study. Taking 3,3′-diethylthiacarbocyanine (Φ_F_ = 0.05 in ethanol [16], Φ_F_ = 0.045 in methanol [17] and Φ_F_ = 0.042 in chloroform, this work) as standard, this effect was quantified and data are summarized in Table 1, in air equilibrated samples (similar results were obtained for degassed solution samples) and at room temperature 20 ± 1 °C.

Apparently, the increase in the alkyl length of the 3,3′-substituents only slightly increase Φ_F_ going from methyl to ethyl and propyl. Hexyl and decyl do not provide a further increase in Φ_F_, perhaps even a slight decrease could be detected here.

**Figure 4:**
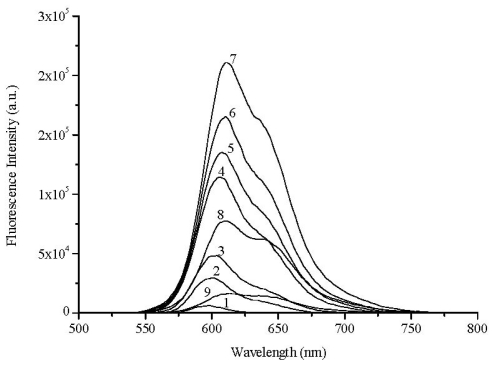
Corrected fluorescence emission spectra of 3,3′-diethylselenocarbocyanine iodide adsorbed onto microcrystalline cellulose for (1) 1 × 10^−8^, (2) 5 × 10^−8^, (3) 1 × 10^−7^, (4) 2 × 10^−7^, (5) 3 × 10^−7^, (6) 5 × 10^−7^, (7) 1 × 10^−6^, (8) 5 × 10^−6^, (9) 1 × 10^−5^ mol of dye per gram of substrate.

Figure 4 shows the laser induced fluorescence of 3,3′-diethylselenocarbocyanine adsorbed onto microcrystalline cellulose for increasing loadings of the dye, going from 1 × 10^−8^ to 5 × 10^−6^ moles of the dye per gram of cellulose using an excitation wavelength of 337 nm.

For dyes adsorbed onto microcrystalline cellulose, it is possible to correlate the fluorescence intensity, measured as the total area under the emission spectra (*I*_F_) as a function of the light absorbed by the dye at the excitation wavelength. *I*_F_ depends on the amount of light absorbed by the dye according to:

*I*_F_ = *S* × *I*_0_ × (l – *R*_exc_) × *f*_dye_ ×Φ_F_, where *S* is a geometrical factor, *I*_0_ is the excitation intensity at the excitation wavelength, *R*_exc_ is the reflectance measured at the excitation wavelength*, f*_dye_ the fraction of the excitation light absorbed by the dye at the excitation wavelength and Φ_F_ is the fluorescence quantum yield of the adsorbed dye [5,11]. This simple relation holds whenever the emissive species is in the monomeric form, (low loadings of the dye) and self-absorption does not play a too important role [18]. For larger loadings the H aggregated forms of the dye starts to absorb and a strong decrease in *I*_F_ was observed, as sample 8 of Figure 4 clearly shows. *f*_dye_ changes with wavelength for high loadings, because it includes monomer and dimer absorption. Therefore Φ_F_, the fluorescence quantum yield of the adsorbed dye, was determined only for low loadings where only monomers exist (although a calculation including the aggregated forms of the dye could easily be performed, if one is interested in the emission of the aggregates).

We have also determined Φ_F_ with the use of the integrating sphere for low and high levels of dye loading for the powdered samples under study. In many cases Φ_F_ does not change until concentrations of ~1 μmol per gram are reached. However, when we consider the influence of the alkyl chain in the two series of cyanines, a decrease of Φ_F_ with the increase of the length of the alkyl chain was observed, and is presented in Table 2.

### 2.3. Quantum Yield of Singlet Oxygen

**Figure 5:**
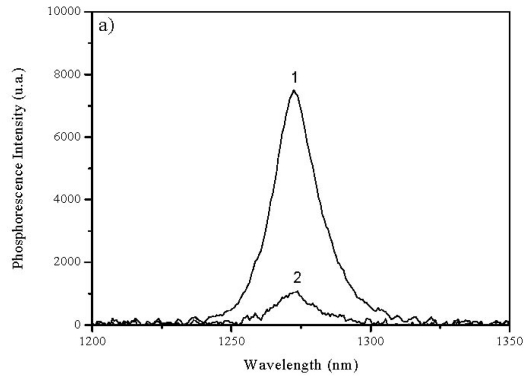

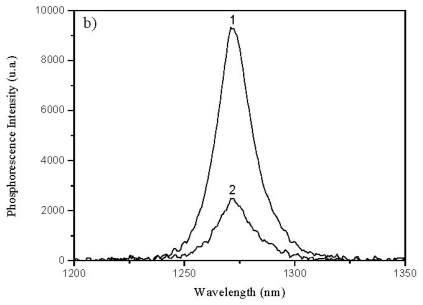
Singlet Oxygen Emission Spectra of: (**a**) 1: Phenazine, 2: 3,3′-dimethylthiacarbocyanine iodide and (**b**) 1: Phenazine, 2: 3,3′-dimethylselenocarbocyanine iodide in chloroform. The optical density at the excitation wavelength (337 nm) was 0.3 in all cases.

Singlet oxygen studies of the two series of cyanine dyes in chloroform were performed and some of the results obtained are presented in Figure 5, both for standard (Phenazine in chloroform Φ_F_ = 0.84) and sample under study. The Table 3 shows the obtained quantum yields for all dyes in chloroform.

These findings are considered to be a consequence of the steric hindrance felt by the adsorbates which bear long chains substituents, forcing deviations from planarity, therefore reducing the radiative processes of deactivation. The Φ_Δ_ values for the selenocarbocyanine family are larger than those of the thiacarbocyanine family for the same alkyl substituent, certainly due to the increase of intersystem crossing rate due to the enlargement of the heavy atom effect when compared to the thia compounds.

### 2.4. Lifetime Distribution Analysis

Recently we developed a new tool for lifetime distributions analysis (LDA) of emissions of probes adsorbed onto heterogeneous surfaces [19]. This new methodology uses pseudo-Voigt profiles (Gaussian-Lorentzian product) instead of pure Gaussian or Lorentzian distributions and allows for asymmetric distributions. Microsoft Excel Solver was the tool used for the fitting procedures. LDA proved to be a very convenient way to treat the phosphorescence or fluorescence decay data because it reflects the multiplicity of sites available for the probe molecules on the specific surface under study [19,20]. The use of a sum of several exponentials to analyse the decay of probes onto heterogeneous surfaces is a description without physical meaning [19,20]. The validation of the conclusions of the LDA analysis should be simultaneously sustained by other spectroscopic studies.

In this work, and due to the quite short solution lifetimes of the thia and seleno carbocyanines, we used the software provided by the EasyLife manufacturer which enables lifetime evaluation with accuracy, starting at about 90 ps, up to 3 μs. The fluorescence decay is approximated by a sum of exponential series weighted by variable amplitudes. No specific profiles for the amplitudes’ determination are used here, and the amplitudes and lifetimes are obtained by minimizing the chi-square function where the excitation pulse profile and the instrumental response function are taken into account in the convolution matrix.

**Figure 6:**
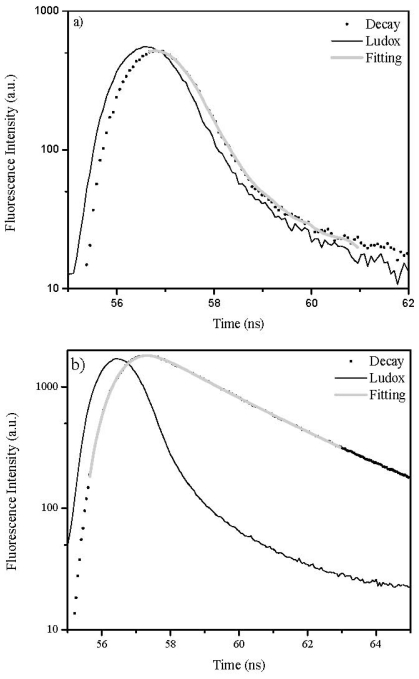
Fluorescence decay (·) and lifetime analysis (−) for 3,3′-dimethylthiacarbocyanine dyes in: (**a**) chloroform and **(b**) adsorbed onto microcrystalline cellulose using a 310 nm LED as a pulsed light source.

Figure 6 presents a comparison between solution sample decays and microcrystalline samples of the 3,3′-dimethylthiacarbocyanine dye. A remarkable increase of lifetimes was observed for the solid powdered samples, in accordance with a remarkable decrease of the non-radiative deactivation processes, in this case a decrease in the photoisomerization process, the most important one for cyanines.

**Figure 7:**
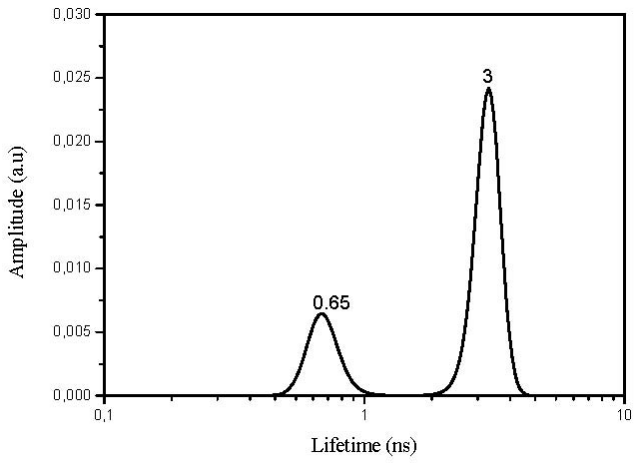
Exponential Series Method (ESM) analysis of dye 10 adsorbed on microcrystalline cellulose. The sample concentration was 1 × 10^−5^ mol of dye per gram of substrate.

Figure 7 shows some of the results obtained with the use of LDA for the case 3,3′-dimethylthiacarbocyanine dyes adsorbed onto microcrystalline cellulose.

Both laser-induced room temperature time resolved fluorescence spectra and fluorescence lifetime measurements with the use of a lifetime distribution analysis for the previously referred solid powdered samples revealed, in most cases, the existence of double emissions with lifetimes at least one order of magnitude above that obtained for the solution studies (see Table 4). In the powdered solid samples always two fluorescence lifetimes are identified accounting for the different locations of the dyes on the cellulosic environment.

These results suggest that the dyes are emitting in two different environments. One very much ordered, where the cyanines are well entrapped into the cellulose polymer chains, previously swelled by the use of a protic and polar solvent, ethanol in this case. In this environment, the cyanines exhibit the largest fluorescence lifetime due to the high constrain imposed by the entrapment, resulting in the decrease of the non-radiative pathways of deactivation namely the photoisomerization process.

In contrast with the slower component, the faster decays point to a more flexible environment. Therefore, the distribution at shorter lifetimes is assigned to cyanines located in more disorganized, *i.e.*, more amorphous regions of cellulose. In this less constrained environment, adsorption sites are characterized by interactions with stereochemically available cellulose hydroxyl groups, enabling radiationless deactivation of the excited state namely through photoisomerization, leading to shorter emission lifetimes.

### 2.5. High Fluences of Laser Excitation

**Figure 8:**
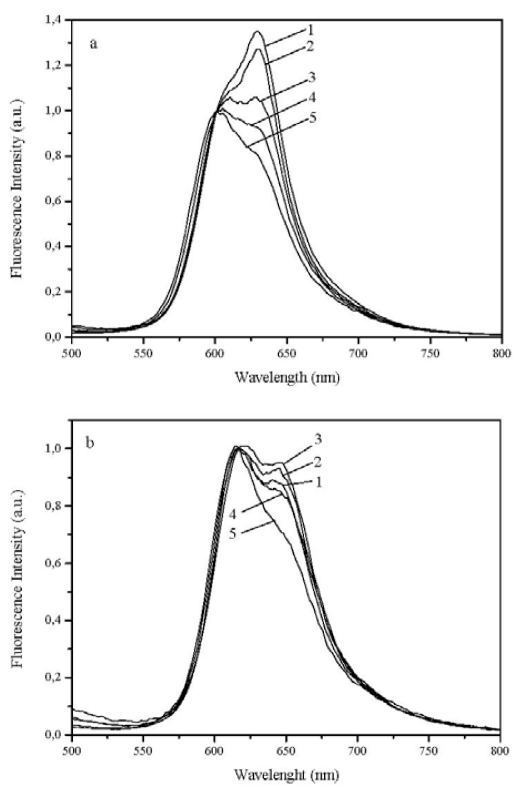
(**a**) Corrected fluorescence emission spectra of dyes 1, 2, 3, 4 and 5 adsorbed onto microcrystalline cellulose (1 × 10^−6^ mol of dye per gram of substrate) normalized at 601 nm, and (**b**) corrected fluorescence emission spectra of dyes 6, 7, 8, 9 and 10 adsorbed onto microcrystalline cellulose (1 × 10^−6^ mol of dye per gram of substrate) normalized at 601 nm. In all cases the excitation wavelength was 337 nm.

Figure 8a shows the fluorescence emission of 1 μmol·g^−1^ samples of dyes 1 to 5, onto powdered solid samples of microcrystalline cellulose. Clearly the photoisomer P emission is now well seen (peaking at about 630 nm), contrary to the emissions with low fluences at the same excitation wavelength where the *N* species emits with a maximum at about 600 nm for the thia compounds, as reported by us before for 1,1′-diethyl-2,2′-carbocyanines [5,15]. However, going into longer *n*-alkyl chain length, *i.e.*, in the series methyl, ethyl, propyl, hexyl or decyl chains, the photoisomer emission is systematically reduced, certainly because the photoisomerisation process is inhibited by steric hindrance imposed by the increasing length alkyl substituent. A very similar pattern was observed in the selenocarbocyanines series, probably because in this case, the importance of photoisomerization is reduced because of the increase of intersystem crossing efficiency due to heavy atom effect of the selenium atom.

## 3. Experimental Section

### 3.1. Materials

Cyanine dyes **2** and **3** were purchased from Aldrich and used without further purification. All other dyes were prepared by condensing the appropriate 2-methylbenzoazolium iodide with triethyl orthoformate in the presence of pyridine, according to the literature procedure [21]. All synthesized compounds showed spectral data fully consistent with the assigned structures.

Ethanol and Chloroform (Merck, Uvasol grade) were used as received. Microcrystalline cellulose (Fluka DSO) with 50 mm average particle size was used.

### 3.2. Sample Preparation

Solutions of all dyes were prepared in chloroform. Solid powdered samples were made by the use of ethanol, and by the solvent evaporation methodology described elsewhere [10,14], from suspensions of microcrystalline cellulose in ethanol.

Cyanines adsorption onto microcrystalline cellulose were achieved by the solvent evaporation method, which consists in the addition of an ethanolic solution of the probe (the solvent, ethanol, was previously dried with molecular sieves) to the previously very well dried powdered solid substrate (48 h heating at about 100 °C under *ca.* 10^−2^ Torr vacuum), followed by slow solvent evaporation from the stirred slurry in a fume cupboard. Final solvent removal was performed for at least 12 h in an acrylic chamber with an electrically heated shelf (Heto, Model FD 1.0–110) with temperature control (30 ± 1 °C) and under moderate vacuum (*ca.* 10^−3^ Torr).

### 3.3. Ground State Diffuse Reflectance Absorption Spectra (GSDR)

Ground-state absorption studies of all solid powdered samples were performed using an OLIS 14-VIS-NIR spectroscopy operating system with a diffuse reflectance attachment (90 mm diameter integrating sphere, internally coated with MgO). A short-wave-pass filter (Corion 600S) was interposed between the sample and the detector (Hammamatsu R955) to reduce the amount of fluorescence emission reaching the detector as much as possible. The calibration of the system was achieved by using a “perfect” reflector (reflectance, *R* = 1.00 from MgO, Aldrich, 99.999%) and a black standard, carbon black, with very finely divided particles (Cabot 2000) which gave *R* = 0. The reflectance, *R*, from each sample was obtained by scanning the excitation monochromator from 240 to 800 nm, and the remission function, *F*(*R*), was calculated using the Kubelka-Munk equation for optically thick samples (those where no further increase of the sample thickness can change the experimentally determined *R*). The remission function is *F*(*R*) = (1 – *R*)^2^/2*R*. Details regarding the data treatment can be found in [11] and references therein.

Steady-state absorption spectra was recorded with the use of a Camspec M501 single beam scanning UV/Visible spectrophotometer at room temperature and in the spectral range of 290 to 800 nm. All the solutions were adjusted to 0.3 absorbance at 337 nm using a UV quartz cell (1 cm path length) for singlet oxygen formation quantum yield.

### 3.4. Absolute and Relative Fluorescence Emission Quantum Yield Determinations

An integrating sphere for relative and absolute measurements was used in this work, as a way to double check the values obtained for the fluorescence emission quantum yields (Φ_F_) of the powdered samples and solutions of the cyanines under study, as EPA recommends [22]. The use of an integrating sphere enables one to measure the absolute Φ_F_’s of virtually any kind of materials, from powders to films and solutions [22–24]. Absolute methods are convenient ways towards the determination of Φ_F_’s, and result in a better approach than the exclusive reliance on a luminescence standard for Φ_F_ calculation [24].

Several methods have been proposed for the measurement of the absolute photoluminescence efficiency, using an integrating sphere to collect the emitted light (hollow sphere, coated in the inside with a diffuse reflecting material, typically, barium sulphate, magnesium oxide, or thermoplastic resins) [22–24].

Fluorescence emission quantum yield (Φ_F_) calculations here described have followed a literature procedure based on an absolute method [22–24]. Using this method, the φ_F_ determination of a standard fluorophore, rhodamine 101 in ethanol (Rod 101), with a known quantum yield, was performed for validation purposes (in ethanolic solution Φ_F_ = 0.91 ± 0.05 in argon saturated samples or air-equilibrated conditions). The good agreement found between the Φ_F_ obtained here by the absolute method and the reported literature values [25] validate the photoluminescence quantum yields determined here for the powdered samples of the cyanines adsorbed onto microcrystalline cellulose.

The fluorescence quantum yields were obtained by the use of the following equations [22–24]:

$$\Phi_{\text{F}}=(P_{\text{c}}-(1-A)\times P_{\text{b}})/A\times L_{\text{a}}\;\text{with }A=(1-L_{\text{c}}/L_{\text{b}})$$

where *A* is the absorption coefficient, *P*_b_ is the light emitted by the sample after absorption of scattered excitation light, *P*_c_ is the light emitted by the sample after absorption of total laser light, *L*_a_ is the total amount of excitation laser light, *L*_b_ is the scattered laser light, and *L*_c_ is excitation light spectrum.

In many cases *P*_b_ is negligible and the equation simply becomes [16]:

$$\Phi_{\text{F}}=P_{\text{c}}/(L_{\text{a}}-L_{\text{c}})$$

### 3.5. Fluorescence Lifetime Determinations

Fluorescence lifetimes were determined using Easylife V™ equipment from OBB (Lifetime range from 90 ps to 3 μs). This technique uses pulsed light sources from different LEDs (310 nm in this case) and measures fluorescence intensity at different time delays after the excitation pulse. In this case a 590 nm cut-off filter was used at emission both for solution and for solid samples. The instrument response function was measured using a Ludox scattering solution. FelixGX software from OBB was used for fitting and analysis of the decay dynamics, 1 to 4 exponentials and also a lifetime distribution analysis, the Exponential Series Method (ESM).

### 3.6. Laser-Induced Luminescence (LIL)

Schematic diagrams of the LIL system were presented in [11]. A N_2_ laser (PTI model 2000, *ca.* 600 ps FWHM, ~1.0 mJ per pulse, and approximately 1 cm^2^ of excitation area) was used in the laser-induced luminescence experiments, the excitation wavelength being 337 nm. With this set-up, fluorescence and phosphorescence spectra were easily available (by the use of the variable time gate width and start delay facilities of the ICCD).

The light arising from the irradiation of solid samples by the laser pulse was collected by a collimating beam probe coupled to an optical fibre (fused silica) and detected by a gated intensified charge coupled device Andor ICCD, model i-Star 720. The ICCD was coupled to a fixed compact imaging spectrograph (Andor, model Shamrock 163). The system can be used either by capturing all light emitted by the sample or in a time-resolved mode. The ICCD has high speed gating electronics (about 2.3 ns) and intensifier. It covers at least the 250–950 nm wavelength range. Time-resolved emission spectra are available in a time range from nanoseconds to seconds.

High fluence laser excitation was obtained by the use of a converging silica lens (100 mm focal distance) which focused the laser excitation beam into a ~2 mm^2^ spot (about fifty times decrease of the normal excitation area).

### 3.7. Quantum Yield of Singlet Oxygen Determinations

The singlet oxygen measurement set-up was assembled in our laboratory. As an excitation source we used the nitrogen laser described in Section 3.6. The detector is an InGaAs CCD (model i-Dus from Andor) working at low temperature (−60 °C) coupled to a fixed spectrograph, model Shamrock 163i, also from Andor. Long pass filters were used to exclude totally avoid the excitation radiation from reaching the detector (LFP1000 or LFP1100 from CVI Lasers).

## 4. Conclusions

Ground state absorption and fluorescence emission studies of 3,3′-dialkyl thia and selenocarbocyanine families of dyes, both in solution and adsorbed onto microcrystalline cellulose were made, and the influence of the alkyl chain length was evaluated in the two media. Simultaneously, fluorescence lifetimes and singlet oxygen quantum yields of formation for the same systems were performed in chloroform solutions.

All solution data support the decrease of the non-radiative mechanisms of deactivation when the alkyl chain is increased from methyl to propyl substituents. However an increase was observed for hexyl and decyl substituents, due to steric hindrance effects.

No direct influence of the alkyl chain length was observed for adsorption of these dyes onto microcrystalline cellulose in terms of the lifetime analysis (two distribution modes), however fluorescence quantum yields exhibit a totally different behaviour: the smaller the substituent, the higher the Φ_F_. These results are interpreted in terms of the steric hindrance felt by the adsorbates which bear long chains substituents, forcing deviations from planarity and consequently reducing the radiative processes of deactivation.

## Figures and Tables

**Table 1:** Fluorescence quantum yields of all dyes in chloroform, using the dye 1 as standard.

Dye	Φ_F_ in CHCl_3_	Dye	Φ_F_ in CHCl_3_
1 (std.)	0.042	1 (std.)	0.042
1	0.029	6	0.023
2	0.042	7	0.038
3	0.046	8	0.044
4	0.046	9	0.033
5	0.040	10	0.034

**Table 2:** Absolute Fluorescence quantum yields of all dyes adsorbed onto microcrystalline cellulose.

Dye	Φ_F_ abs	Dye	Φ_F_ abs
1	0.99	6	0.55
2	0.73	7	0.54
3	0.80	8	0.35
4	0.67	9	0.38
5	0.53	10	0.21

**Table 3:** Singlet oxygen quantum yields of all dyes in chloroform, using phenazine as standard.

Dye	Φ_Δ_	Dye	Φ_Δ_
Phenazine	0.84	Phenazine	0.84
1	0.11	6	0.23
2	0.14	7	0.21
3	0.15	8	0.25
4	0.10	9	0.21
5	0.12	10	0.21

**Table 4:** Fluorescence Lifetimes for 3,3′-dialkylthia and selenocarbocyanine dyes in chloroform and onto microcrystalline cellulose.

Dye	τ_solution_ (ns)	*χ*^2^	τ_1 (solid)_ (ns)	τ_2 (solid)_ (ns)	*χ*^2^
1	0.20	1.80	3.78	0.53	1.56
2	0.32	1.11	3.7	0.56	1.49
3	0.39	1.17	3.53	0.84	1.04
4	0.35	1.59	3.37	0.82	1.15
5	0.31	0.83	3.22	0.43	1.14
6	0.20	1.27	3.45	0.74	1.14
7	0.30	1.89	4.55	0.10	1.39
8	0.33	1.58	3.37	0.71	1.38
9	0.27	1.52	3.07	0.57	1.43
10	0.27	1.60	3	0.65	1.06
